# Supplementary material for: STAT3 Protein–Protein Interaction Analysis Finds P300 as a Regulator of STAT3 and Histone 3 Lysine 27 Acetylation in Pericytes
Source: Biomedicines. 2024 Sep 14;12(9):2102. doi: 10.3390/biomedicines12092102 (PMC11428717; doi:10.3390/biomedicines12092102)
Supplement: Supplementary file 1 [file biomedicines-12-02102-s001.zip › biomedicines-3156671-suppl.pdf]

Supplementary data:

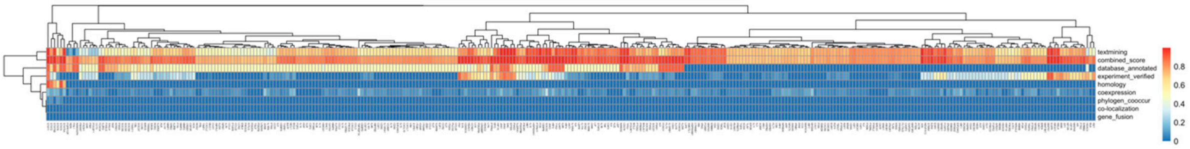

Supplementary Figure S1: Heat map showing 324 STAT3 interacting proteins. A list of 324 proteins with high confidence (total score) interacting with STAT3 is shown as a heat map. The combined confidence score is based on the individual confidence score associated with each type of evidence supporting the interaction.

Most of these proteins are predicted as interactors based on automated text-mining, which inflates the overall score. Text mining is well known to be noisy, hence creating many false positives. The “neighborhood_on_chromosome,” “phylogenetic_cooccurrence,” and “gene_fusion” don’t provide any protein based on this evidence alone.

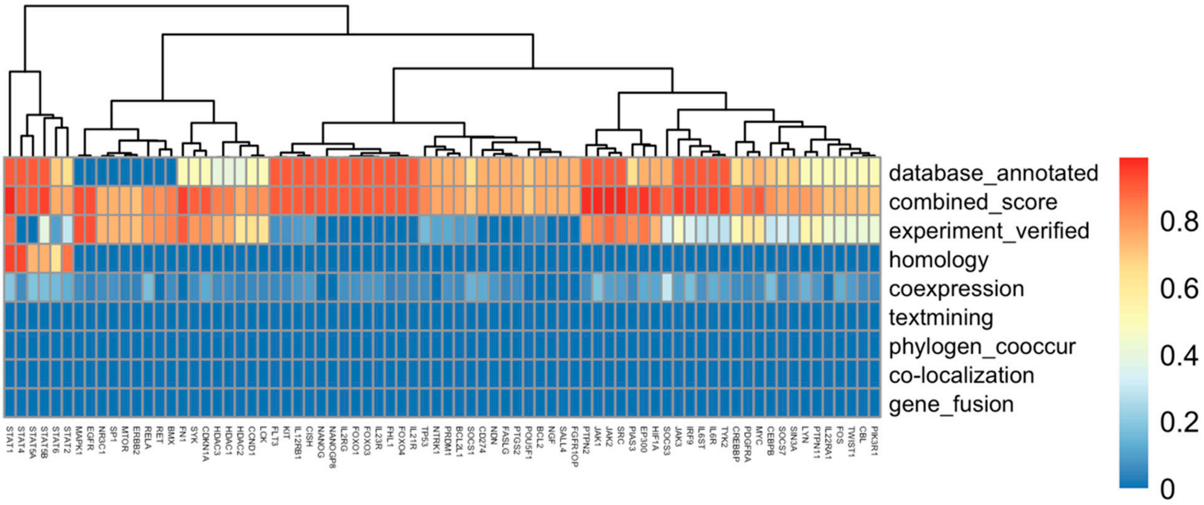

Supplementary Figure S2: Heat map showing 76 STAT3 interacting proteins after applying the filtration criteria. Following filtration criteria, a list of 76 proteins as STAT3 interactors, which shows multiple types of evidence with high confidence, is shown.

STRING-DB input
- Organism: *Homo sapiens*
- Input protein: STAT3

STRING-DB Settings
- Network type: Full network
- Active interaction sources:
  - Experiment
  - Databases
  - Co-expression
- Interaction score: 0.7
- Max interactors to show: 500

STRING-DB network
- Total proteins 77
- Total interactions 377

STRING-DB clustering
- k-means clustering
- No. of clusters: 3

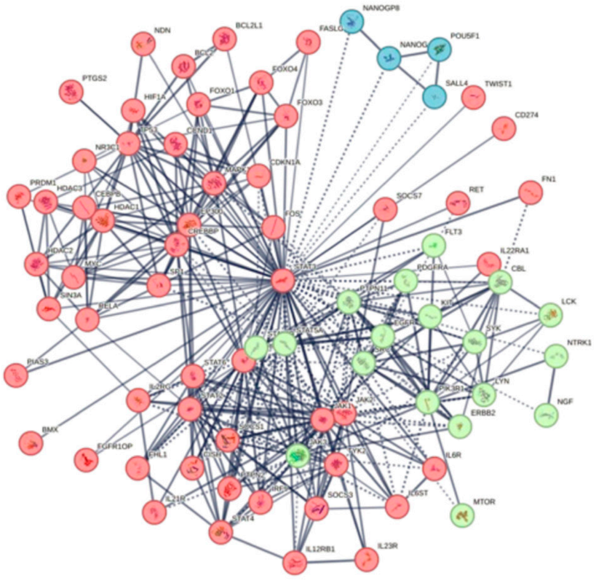

Cluster 1 (Red, 55 proteins): BCL2, BCL2L1, BMX, CCND1, CD274, CDKN1A, CEBPB, CISH, CREBBP, EP300, FASLG, FGFR1OP, FHL1, FN1, FOS, FOXO1, FOXO3, FOXO4, HDAC1, HDAC2, HDAC3, HIF1A, IL12RB1, IL21R, IL22RA1, IL23R, IL2RG, IL6R, IL6ST, IRF9, JAK1, JAK2, MAPK1, MYC, NDN, NR3C1, PIAS3, PRDM1, PTGS2, PTPN2, RELA, RET, SIN3A, SOCS1, SOCS3, SOCS7, SP1, STAT1, STAT2, STAT3, STAT4, STAT6, TP53, TWIST1, TYK2

Cluster 2 (Green, 18 proteins): CBL, EGFR, ERBB2, FLT3, JAK3, KIT, LCK, LYN, MTOR, NGF, NTRK1, PDGFRA, PIK3R1, PTPN11, SRC, STAT5A, STAT5B, SYK

Cluster 3 (Blue, 4 proteins): NANOG, NANOGP8, POU5F1, SALL4

Supplementary Figure S3: STAT3 interactors network for humans based on multiple active sources of interactions. The left panel shows STRING parameters and cutoffs. The middle panel shows the interaction network. Three colors (Red, Blue, and Green) represent each cluster. The list of interacting proteins is depicted in the right panel.

STRING-DB input
- Organism: *Mus musculus*
- Input protein: STAT3

STRING-DB Settings
- Network type: Full network
- Active interaction sources:
    - Experiment
    - Databases
    - Co-expression
- Interaction score: 0.7
- Max interactors to show: 500

STRING-DB network
- Total proteins 43
- Total interactions 192

STRING-DB clustering
- k-means clustering
- No. of clusters: 1

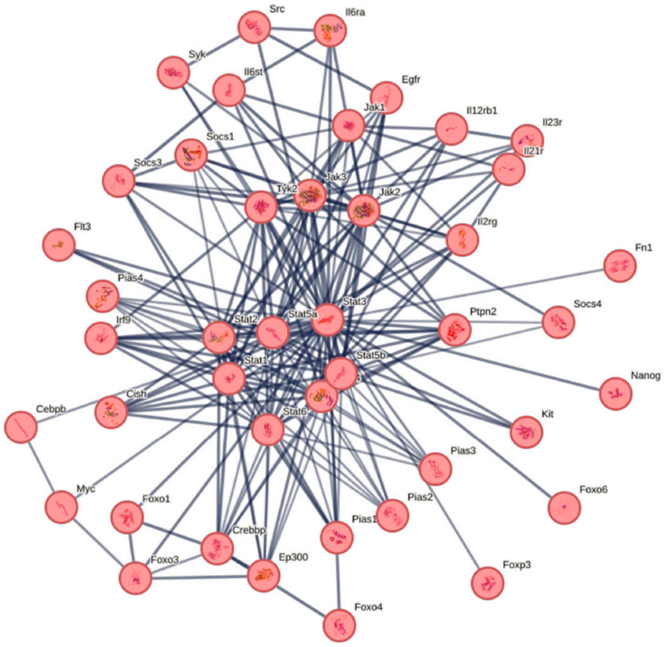

Cluster 1 (Red, 43 proteins): CEBPB, CISH, CREBBP, EGFR, EP300, FLT3, FN1, FOXO1, FOXO3, FOXO4, FOXO6, FOXP3, IL12RB1, IL21R, IL23R, IL2RG, IL6RA, IL6ST, IRF9, JAK1, JAK2, JAK3, KIT, MYC, NANOG, PIAS1, PIAS2, PIAS3, PIAS4, PTPN2, SOCS1, SOCS3, SOCS4, SRC, STAT1, STAT2, STAT3, STAT4, STAT5A, STAT5B, STAT6, SYK, TYK2

Supplementary Figure S4: STAT3 interactors network for mice based on multiple active sources of interactions. The left panel shows STRING parameters and cutoffs. The middle panel shows the interaction network. The red color represents one cluster. The list of interacting proteins is depicted in the right panel.

STRING-DB input
- Organism: *Oryctolagus cuniculus*
- Input protein: STAT3

STRING-DB Settings
- Network type: Full network
- Active interaction sources:
    - Experiment
    - Databases
    - Co-expression
- Interaction score: 0.7
- Max interactors to show: 500

STRING-DB network
- Total proteins 32
- Total interactions 163

STRING-DB clustering
- k-means clustering
- No. of clusters: 2

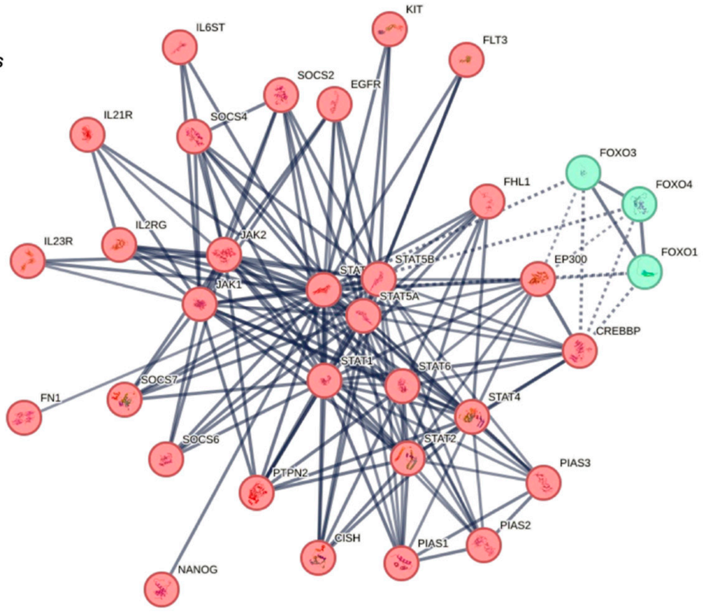

Cluster 1 (Red, 30 proteins): CISH, CREBBP, EGFR, EP300, FHL1, FLT3, FN1, IL21R, IL23R, IL2RG, IL6ST, JAK1, JAK2, KIT, NANOG, PIAS1, PIAS2, PIAS3, PTPN2, SOCS2, SOCS4, SOCS6, SOCS7, STAT1, STAT2, STAT3, STAT4, STAT5A, STAT5B, STAT6

Cluster 2 (Green, 3 proteins): FOXO1, FOXO3, FOXO4

Supplementary Figure S5: STAT3 interactors network for rabbits based on multiple active sources of interactions. The left panel shows STRING parameters and cutoffs. The middle panel shows the interaction network. Two colors (Red and Green) represent each cluster. The list of interacting proteins is depicted in the right panel.

Reactome database

pathway	description	count in network	strength	false discovery rate
HSA-449147	Signaling by Interleukins	46 of 453	1.41	1.39e-50
HSA-1280215	Cytokine Signaling in Immune system	49 of 706	1.25	2.29e-47
HSA-6785807	Interleukin-4 and Interleukin-13 signaling	29 of 107	1.84	6.15e-41
HSA-9006934	Signaling by Receptor Tyrosine Kinases	36 of 521	1.25	1.15e-32
HSA-168256	Immune System	53 of 1979	0.84	4.73e-32
HSA-162582	Signal Transduction	57 of 2540	0.76	1.21e-31
HSA-5663202	Diseases of signal transduction by growth factor receptors ...	32 of 430	1.28	1.22e-29
HSA-1643685	Disease	45 of 1702	0.83	1.76e-25
HSA-201556	Signaling by ALK	14 of 27	2.12	4.34e-22
HSA-5683057	MAPK family signaling cascades	22 of 322	1.24	9.70e-19
HSA-8854691	Interleukin-20 family signaling	12 of 25	2.09	1.76e-18
HSA-1433557	Signaling by SCF-KIT	13 of 43	1.89	5.13e-18
HSA-9674555	Signaling by CSF3 (G-CSF)	12 of 29	2.02	6.11e-18
HSA-451927	Interleukin-2 family signaling	13 of 44	1.88	6.11e-18
HSA-1257604	PIP3 activates AKT signaling	20 of 266	1.28	8.79e-18
HSA-9701898	STAT3 nuclear events downstream of ALK signaling	10 of 10	2.41	1.11e-17
HSA-1059683	Interleukin-6 signaling	10 of 11	2.37	2.00e-17
HSA-9705462	Inactivation of CSF3 (G-CSF) signaling	11 of 24	2.07	7.27e-17
HSA-5684996	MAPK1/MAPK3 signaling	19 of 283	1.23	4.61e-16
HSA-212436	Generic Transcription Pathway	31 of 1215	0.81	8.77e-16
HSA-9020958	Interleukin-21 signaling	9 of 10	2.36	1.18e-15
HSA-9670439	Signaling by phosphorylated juxtamembrane, extracellular a...	10 of 20	2.11	1.22e-15
HSA-2219528	PI3K/AKT Signaling in Cancer	14 of 105	1.53	2.07e-15
HSA-982772	Growth hormone receptor signaling	10 of 23	2.05	3.30e-15
HSA-512988	Interleukin-3, Interleukin-5 and GM-CSF signaling	11 of 48	1.77	2.81e-14
HSA-1266695	Interleukin-7 signaling	9 of 25	1.96	4.53e-13
HSA-9614085	FOXO-mediated transcription	11 of 65	1.64	4.95e-13

Supplementary Table S1: The list of top STAT3 interacting proteins based on the reactome database.

KEGG database

pathway	description	count in network	strength	false discovery rate
hsa04630	JAK-STAT signaling pathway	38 of 158	1.79	5.34e-54
hsa05200	Pathways in cancer	44 of 515	1.34	5.96e-46
hsa04659	Th17 cell differentiation	21 of 99	1.73	1.50e-27
hsa05161	Hepatitis B	23 of 158	1.57	3.49e-27
hsa05167	Kaposi sarcoma-associated herpesvirus infection	24 of 187	1.52	3.49e-27
hsa04151	PI3K-Akt signaling pathway	26 of 349	1.28	3.81e-24
hsa05203	Viral carcinogenesis	21 of 183	1.47	7.80e-23
hsa04917	Prolactin signaling pathway	16 of 68	1.78	1.16e-21
hsa05162	Measles	18 of 137	1.53	1.86e-20
hsa05169	Epstein-Barr virus infection	19 of 192	1.4	1.49e-19
hsa05206	MicroRNAs in cancer	18 of 159	1.46	1.63e-19
hsa05205	Proteoglycans in cancer	19 of 194	1.4	1.63e-19
hsa04658	Th1 and Th2 cell differentiation	15 of 85	1.65	8.54e-19
hsa04919	Thyroid hormone signaling pathway	16 of 120	1.53	2.32e-18
hsa05165	Human papillomavirus infection	21 of 324	1.22	2.39e-18
hsa05220	Chronic myeloid leukemia	14 of 75	1.68	6.74e-18
hsa01521	EGFR tyrosine kinase inhibitor resistance	14 of 77	1.67	8.82e-18
hsa05235	PD-L1 expression and PD-1 checkpoint pathway in cancer	14 of 87	1.61	3.88e-17
hsa05230	Central carbon metabolism in cancer	13 of 68	1.69	8.78e-17
hsa05160	Hepatitis C	16 of 157	1.42	8.78e-17
hsa05212	Pancreatic cancer	13 of 71	1.67	1.31e-16
hsa05215	Prostate cancer	14 of 97	1.57	1.31e-16
hsa05166	Human T-cell leukemia virus 1 infection	17 of 210	1.32	2.22e-16
hsa05163	Human cytomegalovirus infection	17 of 217	1.3	3.56e-16
hsa05223	Non-small cell lung cancer	12 of 68	1.65	3.25e-15
hsa04066	HIF-1 signaling pathway	13 of 102	1.51	7.54e-15
[illegible]	[illegible]	[illegible]	[illegible]	[illegible]

Supplementary Table S2: The list of top STAT3 interacting proteins based on the KEGG database.

WIKI pathways

pathway	description	count in network	strength	false discovery rate
WP49	IL-2 signaling pathway	19 of 42	2.06	1.71e-29
WP395	IL-4 signaling pathway	20 of 54	1.98	1.71e-29
WP2374	Oncostatin M signaling pathway	20 of 64	1.9	1.01e-28
WP2037	Prolactin signaling pathway	20 of 76	1.83	1.48e-27
WP2034	Leptin signaling pathway	19 of 76	1.81	8.37e-26
WP4666	Hepatitis B infection	22 of 150	1.57	1.02e-25
WP2203	Thymic stromal lymphopoietin (TSLP) signaling pathway	17 of 47	1.97	2.18e-25
WP4538	Regulatory circuits of the STAT3 signaling pathway	18 of 77	1.78	4.30e-24
WP304	Kit receptor signaling pathway	17 of 59	1.87	4.54e-24
WP4659	Gastrin signaling pathway	19 of 113	1.63	3.45e-23
WP4172	PI3K-Akt signaling pathway	25 of 336	1.28	5.18e-23
WP585	Interferon type I signaling pathways	16 of 54	1.88	7.25e-23
WP127	IL-5 signaling pathway	15 of 40	1.98	1.06e-22
WP364	IL6 signaling pathway	15 of 43	1.95	2.45e-22
WP437	EGF/EGFR signaling pathway	20 of 162	1.5	3.18e-22
WP286	IL-3 signaling pathway	15 of 49	1.89	1.13e-21
WP5130	Th17 cell differentiation pathway	16 of 69	1.77	1.55e-21
WP4630	Measles virus infection	18 of 135	1.53	1.70e-20
WP2332	Interleukin-11 signaling pathway	14 of 44	1.91	1.92e-20
WP4806	EGFR tyrosine kinase inhibitor resistance	16 of 83	1.69	1.92e-20
WP5098	T-cell activation SARS-CoV-2	16 of 88	1.67	4.02e-20
WP205	IL-7 signaling pathway	12 of 25	2.09	2.98e-19
WP236	Adipogenesis	17 of 130	1.52	2.98e-19
WP3303	RAC1/PAK1/p38/MMP2 pathway	14 of 68	1.72	3.07e-18
WP2261	Glioblastoma signaling pathways	14 of 82	1.64	3.08e-17
WP61	Notch signaling pathway	13 of 61	1.74	4.25e-17
WP673	ErbB signaling pathway	14 of 89	1.6	8.02e-17
WP138	Androgen receptor signaling pathway	14 of 90	1.6	8.91e-17

Supplementary Table S3: The list of top STAT3 interacting proteins based on the WIKI database.

Supplementary Table S4: The list of total STAT3 interacting proteins in humans analyzed by the KEGG database.

Supplementary Table S5: The list of total STAT3 interacting proteins in humans analyzed by the Reactome pathway database.

Supplementary Table S6: The list of total STAT3 interacting proteins in humans analyzed by the gene ontology biological processes database.
